# Coherent Generation of Photo-Thermo-Acoustic Wave from Graphene Sheets

**DOI:** 10.1038/srep10582

**Published:** 2015-06-08

**Authors:** Yichao Tian, He Tian, Y. L. Wu, L. L. Zhu, L. Q. Tao, W. Zhang, Y. Shu, D. Xie, Y. Yang, Z. Y. Wei, X. H. Lu, Tian-Ling Ren, Chih-Kang Shih, Jimin Zhao

**Affiliations:** 1 Beijing National Laboratory for Condensed Matter Physics and Institute of Physics, Chinese Academy of Sciences, Beijing 100190, China; 2 Institute of Microelectronics & Tsinghua National Laboratory for Information Science and Technology (TNList), Tsinghua University, Beijing 100084, China; 3 Department of Physics, Texas University at Austin, Texas 78712, USA

## Abstract

Many remarkable properties of graphene are derived from its large energy window for Dirac-like electronic states and have been explored for applications in electronics and photonics. In addition, strong electron-phonon interaction in graphene has led to efficient photo-thermo energy conversions, which has been harnessed for energy applications. By combining the wavelength independent absorption property and the efficient photo-thermo energy conversion, here we report a new type of applications in sound wave generation underlined by a photo-thermo-acoustic energy conversion mechanism. Most significantly, by utilizing ultrafast optical pulses, we demonstrate the ability to control the phase of sound waves generated by the photo-thermal-acoustic process. Our finding paves the way for new types of applications for graphene, such as remote non-contact speakers, optical-switching acoustic devices, *etc*.

Since graphene was discovered a decade ago^1^, its remarkable properties have been utilized for novel devices and technological applications^2^,^3^,^4^,^5^,^6^,^7^,^8^,^9^,^10^. The outstanding properties of graphene primarily originate from its Dirac-particle-like electronic states^11^,^12^,^13^,^14^,^15^. Early work focused on its exceptional transport properties employing states near the Dirac point^16^,^17^,^18^,^19^,^20^,^21^. The existence of Dirac-like electronic states over a large energy window also resulted in many novel optical properties spanning across a large frequency range^4^,^10^,^22^. The interplay of its unique electronic structures and lattice vibrations under photo-excitation can also lead to interesting properties, which can be harnessed for energy applications. In the present article, we introduce another innovative application: coherent generation of acoustic waves in ambient environments. Specifically, by using ultrafast laser pulses, we demonstrate the generation of acoustic pulses from a multi-layer graphene (MLG) sheet through a photo-thermo-acoustic (PTA) process. Most intriguingly, we show the phase coherence between the acoustic pulses through the phase interferences between sequentially generated acoustic pulses.

We note that graphene and MLG have already been used for the generation of thermo-acoustic waves (*i.e.* sound waves) in ambient air^23^,^24^,^25^. Nevertheless, these studies used graphene sheets or monolayer graphene solely as sheet resistors, similar to thin metal sheets, for the thermo-acoustic generation of sound waves, rather than employing graphene’s characteristic properties. In the PTA process utilized here, the unique electronic properties of graphene play a key role.

## Results

### Photo-thermo-acoustic wave generation in MLG

**Figure 1:**
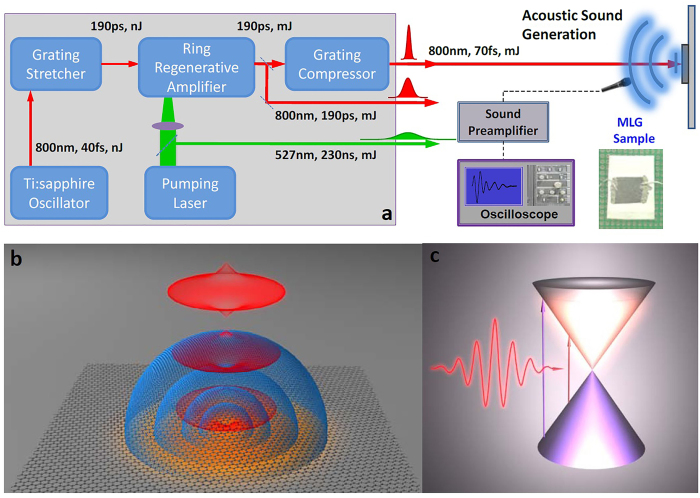
Schematic diagram of the experimental setup and sound generation mechanism. (**a**) Ultrafast laser pulses of different wavelengths, time durations, and repetition rates are irradiated onto the graphene sheet sample. (**b**) Ultrafast laser pulses generate a thermal gradient which leads to acoustic sound wave generation. The time interval between pulses phase-controls the sound amplitude. (**c**) MLG under ultrafast laser pulse excitation. The electron-phonon interaction generates thermal heat during the ultrafast (ps) relaxation process, which further produces acoustic sound at a much longer (μs) time scale. The cones are used to mimic the band structure of MLG.

**Figure 2:**
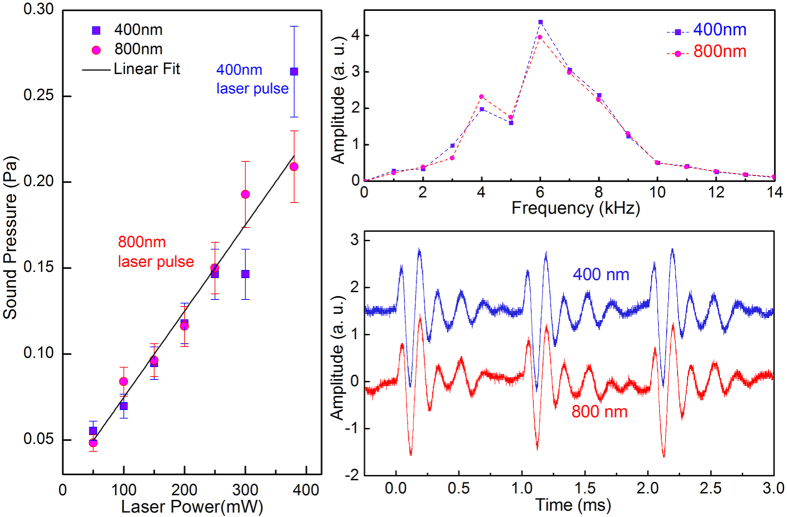
Effect of the photon energy (laser wavelength) on the efficiency of sound generation. The laser pulses with 400 nm and 800 nm central wavelengths have similar effects on the sound generation efficiency in the time domain (with offset), in the frequency domain, and also in intensity (with offset).

**Figure 3:**
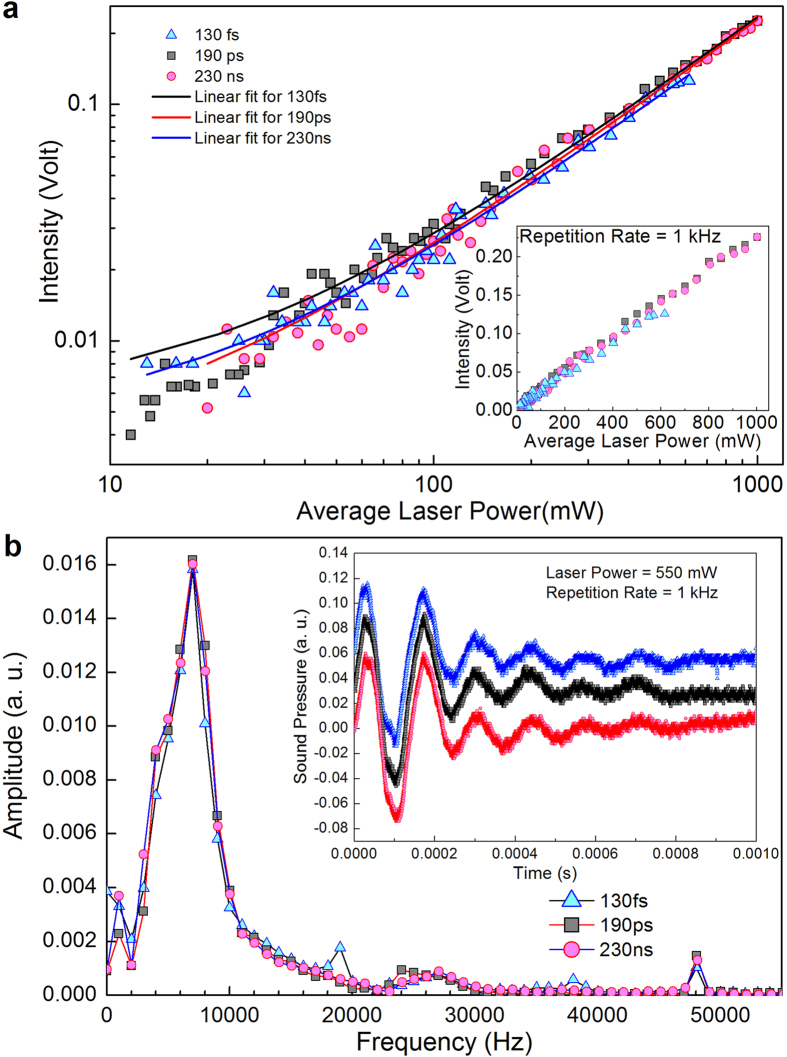
Effect of the laser pulse duration on the sound generation efficiency. (**a**) The blue, black, and red dots indicate the 130 fs, 190 ps, and 230 ns laser pulses, respectively. For a large dynamical range the slope of the three are the same. (**b**) The frequency domain amplitudes and the time domain signals (inset, with offset) of the sound waves, produced with different pulses.

**Figure 4:**
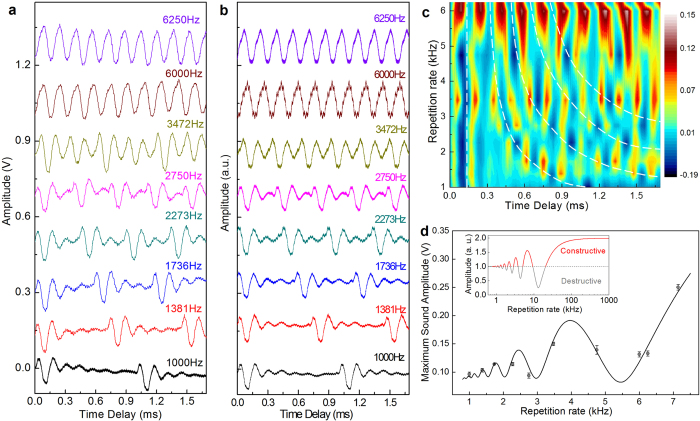
Interference effect and phase control. (**a**) Time domain signal of MLG sound at different laser repetition rates. Additional offset has been applied to the curves for clarity. (**b**) Numerical simulation of the phase control. The superposition of two consecutive acoustic waves gives the well-simulated signal displayed in (**a**). (**c**) False color mapping of the time-domain signal at the different laser repetition rates shown in (**a**). (**d**) Analytical result for the phase control. Constructive and destructive effects are controlled by tuning the repetition rate. The dots are the experimental results shown in (**a**), and the solid curve is a plot of our theoretical equation. The inset shows the result with a fixed average laser power.

The experimental setup is schematically shown in Fig. 1a, which displays the ultrafast laser pulses used to generate and phase-control acoustic sound waves in a MLG sheet sample (see Methods). We note that the electrical contacts seen on the sample in Fig. 1a are used for other experiments and they are not included in a closed circuit. The sound generation is performed through a PTA mechanism, illustrated in Fig. 1b. It comprises a two-stage process in temporal sequence: a photo-thermal (PT) process, followed by a thermo-acoustic (TA) process as described below. When laser pulses illuminate the MLG membrane, the absorbed photons excite the valence band electrons, generating free carriers in the conduction band (electrons) and the valance band (holes). Such excited-state free charge carriers relax to the ground state by emitting optical and acoustic phonons^26^,^27^(Fig. 1c). Extensive interaction between electrons and lattice vibrations leads to a hot lattice temperature (with a radial thermal gradient as shown in Fig. 1b). It has been well-established that the energy relaxation of hot carriers to the hot lattice temperature is mediated by the electron-phonon interaction^28^,^29^,^30^,^31^, leading to an efficient PT process occurring at a relatively fast time scale of the order of picoseconds (see SI1)^11^,^32^,^33^,^34^. The sample then heats the ambient air atoms through vibrations and collisions, which modifies air pressure accordingly^35^, leading to the generation of longitudinal sound waves in the air (Fig. 1b). In essence, this second stage is a TA process^36^. The longitudinal sound waves, which are plain waves in the near field, become spherical waves in the far field (Fig. 1b; for quantitative characterization, see SI2). In this manner, the train of input optical pulses produces a train of acoustic pulses detected in the far field. Most interestingly, each optical pulse generates an acoustic pulse with high (anharmonic) acoustic frequency with a well-defined phase (Fig. 2, 3, 4), which enables us to achieve phase control of the sound generation.

We first investigated the PTA process as a function of the excitation wavelength. We used both 800 nm and 400 nm laser pulses to examine the sound generation efficiency. The experimental results are shown in Fig. 2, which displays the temporal signal trace of the generated acoustic sound. The major peaks are separated by 1 ms, corresponding to the laser pulse repetition rate of 1 kHz, where the laser pulse width is 130 fs. Furthermore, higher frequency oscillations are observed between the major peaks. These faster oscillations correspond to the characteristic frequency of the acoustic sound wave, as we will discuss in the following. In Fig. 2 we show the frequency domain analysis of the corresponding time domain data within one period. It is evident that the time and frequency domain analyses show indistinguishable results for the 800 nm and 400 nm optical excitations. Moreover, the efficiency for sound generation is also independent of the laser wavelength, since the generated sound pressures have the same power dependence (Fig. 2). One can also quantitatively determine a sound generation efficiency of 0.012% (see SI3). This photon-energy-independent feature can be attributed to two factors—the photon-energy-independent absorption coefficient in the visible to the near IR range^37^,^38^, due to a large energy window of the Dirac-like electronic states; and a very efficient energy relaxation channel for the hot electrons (holes) to reach equilibrium with the lattice temperature—both of which have been regarded as hallmarks of the remarkable properties of graphene. The PTA conversion efficiency of 0.012% is nearly identical to the efficiency of the TA process investigated earlier using pure Joule heating (SI3), implying an almost ideal energy conversion efficiency of the photo-thermal process in the MLG sheet.

Subsequently, we investigated the effect of the laser pulse duration on the generation efficiency. Three laser beams with durations of 130 fs, 190 ps, and 230 ns (see Methods) were employed, all at 1 kHz repetition rate. The microphone detection distance was 25 mm. The experimental results are shown in Fig. 3, which demonstrates that within two orders of magnitude dynamic range of the laser power, the slope, and thus the sound generation efficiency, is nearly identical for the three pulse durations. Moreover, the line shape of the acoustic waves is independent of the excitation pulse duration. In Fig. 3b we show the Fourier transform of the time domain data (Fig. 3b inset) that is taken for exactly one period. A peak is clearly observed around 6 kHz. For all three pulse durations, the frequency components and their amplitudes are identical. Unlike the 1 kHz repetition rate observed in Fig. 2 lower right panel, this 6 kHz *anharmonic* signal is more interesting, which has never been reported before. One needs a pulsed excitation source to observe this anharmonic signal. We show that this 6 kHz characteristic frequency originates from the interaction between the sample and the ambient gas molecules. By changing the ambient condition (e.g. using Helium gas in an enclosure) we observed that this 6 kHz frequency changed to ~2 kHz (Figure S2 in SI4).

Considering the results shown in Fig. 3, we were able to ascribe the sound generation to a PTA mechanism, a two-step process comprising an ultrafast PT process followed by a slower TA process. First we eliminated the possibility of a direct photo-acoustic (PA) mechanism. In the PA mechanism, the photo-excited electrons interact directly with the ambient air molecules. The ultrafast dynamics of the free carriers, the phonons, and their interactions all have their characteristic time scales, ranging from tens of femtoseconds to picoseconds to sub-nanoseconds (see SI1). If a direct PA mechanism was involved: (1) the generation efficiency will be higher for the 130 fs pulses, because for 190 ps and 230 ns pulses a prominent portion of the absorbed photon energies are inevitably dissipated through electron-phonon scattering (as thermal energy, instead of acoustic energy); (2) the peak width of the acoustic wave should be smaller for the 130 fs and 190 ps cases, since it is only limited by the ultrafast electron-air molecule scattering rate. This is contrary to our experimental results. The above two reasons are summarized in a table in the supplementary information (see SI5). Our careful experiment in both the temporal and the frequency domain with different pulse widths (Fig. 3) is a direct experimental proof of the PTA mechanism. Our method also applies to other systems of similar materials. The PTA mechanism that we found is in consonant with the photo-thermal-electric (PTE) rather than the photo-voltaic (PV) mechanism in the electronic transport properties of graphene^39^,^40^,^41^. The ultrafast time scale of the PT process effectively creates a delta-function like temperature pulse on the sample. This sharp (in time) temperature pulse generates sound waves at the air/graphene interface, which then propagate through the air and are detected in the far field.

### Coherent phase-control of the PTA sound waves

An interesting aspect of these PTA generated acoustic waves is the well-defined frequency (~6 kHz, different than the laser repetition rate) and the well-defined phase in the time domain. This introduces the interesting prospect of coherently controlling the relative phase between acoustic pulses, leading to constructive or destructive interferences.

In order to investigate this thoroughly, we used laser pulses of 532 nm wavelength, 400 ns duration, and a fixed energy, thus the average laser power increased linearly with the repetition rate. In Fig. 4a we show the time-resolved acoustic waves, which exhibit constructive and destructive interference effects, as a function of the laser repetition rate (for tuning the repetition rate, see Methods). The relative phase between two consecutive acoustic wave packets in the time domain is directly related to the repetition rate. In Fig. 4b we show a numerical simulation of such an interference effect, by taking the acoustic response of a single pulse and applying strictly the wave superposition according to the laser repetition rate. It is evident that the numerical simulations using wave superposition accurately reproduce the experimental results. In Fig. 4c we show the false color mapping of the result in Fig. 4a to clearly illustrate the phase-control effect. Owing to the finite number of discrete values of repetition rates, the interpolation is implemented between the measured data. The phase tuning is marked by white dashed curves and the interference effect is manifested by the horizontal red and blue color stripes. At low repetition rates the interference effect is small, and at relatively high repetition rates the interference becomes more pronounced. The quantitative analysis of such an interference effect is further described in the discussion section. As verified in additional experiment (results not shown here), tuning the repetition rate at much lower than 1000 Hz (for example, from 1 Hz to 1000 Hz) has very little effect on the sound amplitude. However, as the laser repetition rate increases, the sound amplitude displays a pronounced increase and decrease alternately (Fig. 4c,d). This modification can be constructive or destructive, depending on the relative phase between the consecutive acoustic wave packets. In Fig. 4c the red stripe corresponds to constructive interference and the blue stripe to destructive interference.

## Discussion

We furthermore performed analytical analysis of the sound amplitude as a function of varying laser repetition rate. Assuming a sinusoidal function superimposed on a single exponential decay for individual acoustic pulses as *A(P)*sin(ω*t*)exp(−β*t*), the superposition of two consecutive pulses can be expressed as


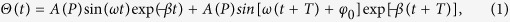


where *T* is the period of the laser pulse repetition (*i.e.* the time interval between two pulses), which is simply controlled by tuning the repetition rate, φ_0_ is a fitting parameter that accounts for the initial additional phase between the two oscillations, *A*(*P*) is the amplitude as a function of the laser power for each acoustic wave packet, and *ω* and *β* are the frequency and decay constants, respectively. Considering proportionality between the laser power, rate, and amplitude, after a simple calculation we derived the interference as


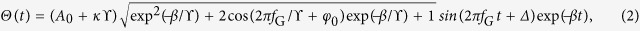


where 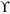 is the laser repetition rate, ƒ_G_ = ω/2π is the graphene’s anharmonic oscillation frequency, 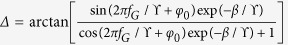, and 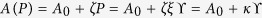, with *ζ*, *ξ*, and *κ* being constant coefficients. The total amplitude of the oscillation Θ(*t*) can thus be controlled by the laser repetition rate as follows:


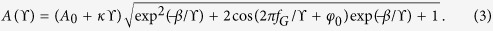


In order to compare with the experimental result, we plotted both the experimental data and the fitted theoretical curve in Fig. 4d. To obtain the experimental amplitude value we have subtracted the minimum amplitude (wave valley) from the maximum amplitude (wave peak) for each curve. The best fitting parameters for the calculation were *A*_0 _= 0.075 V, *κ* *=*  0.0163 V∙kHz^−1^, *ƒ*_G_ = 6.5 kHz, φ_0_ = 1.92, and *β* =3.78 ms^−1^. It is obvious that the theoretical calculation curve compares well with the experimental data (Fig. 4d); therefore this analytical calculation, albeit using harmonic waves of a single frequency, is proved to adequately describe the amplitude as a function of the repetition rate.

In summary, we have demonstrated the precise phase control of acoustic sound wave generation in graphene sheets using ultrafast optical pulses. In the phase control, the constructive and destructive generation efficiency was precisely and easily controlled by tuning the laser repetition rate. Our investigation paves the way to the development of energy applications using graphene materials. Both visible and ultraviolet optical pulses can be used to generate sound waves in graphene sheets, showing the potential for energy harvesting far from the Fermi surface. Anharmonic sound wave generation has been clearly observed and for the first time thoroughly investigated, revealing a PTA physics mechanism. Our work demonstrates an optical sound generation device based on graphene sheets, which has non-contact and remote control capability. Our investigation can be easily extended to electrical interference control and other sound generation applications, such as optical switching of acoustic sound generation.

## Methods

### Sound generation using ultrafast laser pulses

We used multiple ultrafast laser systems as the excitation source. Light pulses with tunable temporal pulse width (70 fs, 130 fs, 190 ps, 230 ns, and 400 ns), repetition rate (0–8 kHz), and photon energy (with 800nm and 400 nm wavelength) were used as excitation sources with a normal incident geometry. The optical beam was expanded to a diameter of 10 mm on the sample surface using a lens system. The sound signal was detected with a microphone and amplifier system and quantitatively recorded with an oscilloscope. When the laser power was increased to 50 mW, acoustic sound could be heard by the ears at 10 cm away from the sample. When the laser beam was blocked, the sound disappeared; as the laser power was increased, the sound volume increased accordingly. To ensure that the sound was produced by the graphene sheets instead of the paper substrate, a control experiment was performed on the bare paper substrate. Under the same conditions and upto the maximum laser power (595 mW for the 130 fs laser beam, 2 W for 190 ps laser beam, and 1.59 W for the 230 ns laser beam), no sound signal was detected. Our MLG sheet on the paper substrate had a 1 × 1 cm^2^ area and an average thickness of 60 nm. The sample (Fig. 1) was fabricated by CVD on Ni, with details described in Ref. 25. The X-ray Diffraction data of our sample is shown in SI6. The electrical contacts seen in Fig. 1 are used for other experiments and they are not included in a closed circuit.

### Acoustic sound wave detection

The sound intensity was detected using a microphone (TM-12, Tong Sheng Inc. http://www.tonsion.com.cn/productInfo.aspx?typeid=23&id=58), whose output was sent into a preamplifier (JX-01B, Ju Long Inc.) before it was input into the oscilloscope (DPO 4000, Tektronix, Inc.), which had a sampling rate of 5 GS/s and bandwidth of 1 GHz. To obtain the data shown in Fig. 4a, the same microphone was placed at a distance of 2.5 cm and an angle of 45° from the sample, in order to collect the sound signal and to convert it into electrical signal; the latter was then amplified by a different preamplifier (KX-2A, Kesuosi Inc.) before being recorded by an oscilloscope (DSO 7104B, Agilent Technologies Inc.).

### Ultrafast Laser System

Our laser system was an in-house built chirped-pulse amplifier (CPA), which consisted of a femtosecond Ti:sapphire oscillator, a pump laser, a stretcher, a ring regenerative amplifier, and a compressor. Initially, stable femtosecond laser pulses as short as 40 fs were generated from the Ti:sapphire oscillator at a repetition rate of 80 MHz. Subsequently, a grating stretcher was used to stretch the pulse duration to 190 ps. Following the stretcher, the laser pulse was injected into the regenerative amplifier, which was pumped by a commercial 527 nm pump laser with a pulse duration of 230 ns at a repetition rate of 1 kHz. By optimizing the time delay between the seeding and pumping pulses, the chirped laser beam was amplified progressively and continually until saturating at the maximum gain before being extracted from the cavity. Finally, the fully amplified chirped laser pulse was compressed to 70 fs by using a single grating compressor after 4-pass diffraction. The typical energy was approximately 3 mJ and the bandwidth was 18.4 nm (FHWM). We utilized the laser from the 527 nm pump laser (230 ns), the amplified pulse before compression (190 ps), and the final compressed pulse (70 fs), respectively, for our experiment. For the pulse duration investigation, the laser pulses were supplied by the chirped-pulse amplifier laser system and the 130 fs commercial laser system.

### Tuning of the repetition rate

Besides the ultrafast laser systems described above and illustrated in Fig. 1, two additional ultrafast systems were used in our experiment. They had a single wavelength, but were able to provide a tunable laser pulse repetition rate. One of them could be tuned from 1 Hz to 1000 Hz and the other from 1000 Hz to 10 kHz. The tuning of the latter one was challenging, because each tuning required opening the laser cavity and re-calibrating the system.

## Additional Information

**How to cite this article**: Tian, Y. *et al.* Coherent Generation of Photo-Thermo-Acoustic Wave from Graphene Sheets. *Sci. Rep.* **5**, 10582; doi: 10.1038/srep10582 (2015).

## Supplementary Material

Supplementary Information
