# Supplementary material for: Coherent Generation of Photo-Thermo-Acoustic Wave from Graphene Sheets
Source: Sci Rep. 2015 Jun 8;5:10582. doi: 10.1038/srep10582 (PMC4650607; doi:10.1038/srep10582)
Supplement: Supplementary Information [file srep10582-s1.doc]

Supplementary Information

**Coherent Generation of Photo-Thermo-Acoustic Wave from Graphene Sheets**

Yichao Tian^1†^, He Tian^2†^, Y. L. Wu^1^, L. L. Zhu^1^, L. Q. Tao^2^, W. Zhang^1^, Y. Shu^2^, D. Xie^2^, Y. Yang^2^, Z. Y. Wei^1^, X. H. Lu^1^, Tian-Ling Ren^2^*, Chih-Kang Shih^3^*, Jimin Zhao^1^*

^1^ *Beijing National Laboratory for Condensed Matter Physics and*

*Institute of Physics, Chinese Academy of Sciences, Beijing 100190, China*

*^2^ Tsinghua National Laboratory for Information Science and Technology, Institute of Microelectronics, Tsinghua University, Beijing 100084, China*

^3^ *Department of Physics, Texas University at Austin, Texas 78712, USA*

^†^ These authors contributed equally to this work

* To whom correspondence should be addressed. E-mail: [rentl@tsinghua.edu.cn](mailto:rentl@tsinghua.edu.cn) (T.R.); [shih@physics.utexas.edu](mailto:shih@physics.utexas.edu) (C.-K.S.); [jmzhao@iphy.ac.cn](mailto:jmzhao@iphy.ac.cn) (J.Z.)

**SI1 Ultrafast carrier dynamics in graphene:**

The ultrafast dynamics of photo-excited carriers in graphene is characterized by several different time scales. Within a few femtoseconds upon light pulse incidence, electrons are coherently excited to the excited states (the time scale of excitation is often limited by the light pulse profile), where the energy above the Fermi level is roughly half of the excitation photon energy for undoped graphene. Subsequently the electron-electron scattering occurs among the excited carriers. After tens of femtoseconds the electrons reach thermal equilibrium among each other, while the electrons and phonons are still at different temperatures so far. Such a nonequilibrium state further relaxes through electron-phonon scattering, accompanied by decaying of electron energy through emission of optical phonons. This inelastic scattering lasts hundreds of femtoseconds before an equilibrium state is reached between the electrons and the optical phonons. Thus a uniform temperature for both electrons and optical phonons sets in within one picosecond after excitation. The subsequent cooling for the electrons is strictly limited by the finite density of optical phonons generated, which could only gradually diminish through anharmonic decay into acoustic phonons or/and leaving the active area (although other processes may also take part in), known as the phonon-bottleneck effect. Due to the very large optical phonon energy characteristic for graphene, this bottleneck effect is especially prominent in all graphene materials and devices. It corresponds to a very slow anharmonic decay into acoustic phonons, of which the timescale is tens of picoseconds at room temperature and hundreds of picoseconds below 50K. Ultimately the shared temperature of the equilibrium state is determined by the laser power absorbed and the heat dissipated.

**SI2 Sound generation characterization:**

The laser-power-dependence of the sound intensity was measured and presented in Fig. S1a. The sound intensity is proportional to the laser power, with a linear range covers from a few mW to 1 W laser power. The threshold for detecting sound signals was observed to be 26 mW. The distance-dependence was measured and shown in Fig. S1b. It is evident that the signal decays with increasing distance, with an inverse-dependence relation. In Fig. S1c & d we show the angle-dependences of the sound intensity. Both of the dependences in the vertical plane and in the horizontal plane were measured, where the laser power was kept at 800mW and the measuring distance was 10 mm.

For the Photo-thermo-acoustic (PTA) mechanism we proposed in the article, the sound generation equation can be written as

, (Eq. S1)

where $P_{rms}$ is the root-mean-square sound pressure, $R_{0}$ is the Rayleigh distance, $r_{0}$ is the distance of the microphone from the source, $\gamma$, $\upsilon_{g}$, and $e_{g}$ are the heat capacity ratio, sound velocity, and thermal effusivity of the ambient gas, respectively, $e_{s}$ is the thermal effusivity of the substrate, *M* and $a_{c}$ are the thermal factors related to the thermal conductivity, capacity, density, *etc.*, $P_{in}$ is the average incident laser power, $T$ and $N$ are the transmittance and number of graphene sheets, $\theta$ and are the spherical angles for detection, $k_{0}=2\pi/\lambda_{0}$ is the isotropic sound wave-number, $L_{x}$ and $L_{y}$ are the effective length and width of the sound source, respectively. The counterpart of this equation for the electrical-thermal-acoustic (ETA) mechanism is given in Ref. 23. From Eq. S1 we can see that the sound pressure is linearly dependent on the input power, which is in good agreement with the experimental results in Fig. S1a. Also it is proportional to the inverse of the measuring distance as $P_{rms}\propto1/r_{0}$, which is in agreement with Fig. S1b. The theoretical results of the angle-dependences of the sound pressure are plotted in Fig. S1c & d, respectively, which again compare well with the experimental results.

**
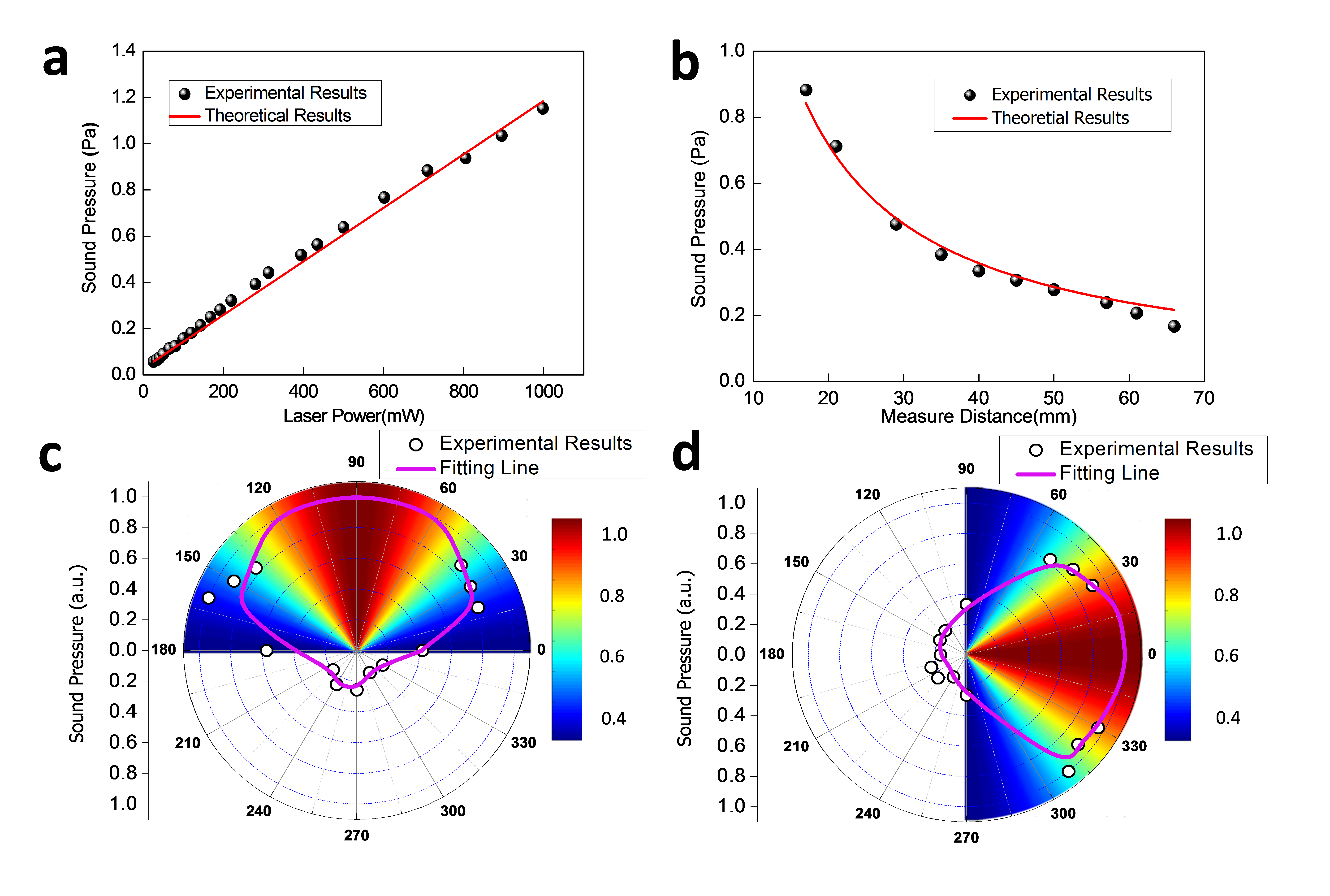
**

**Fig. S1. Power-, distance-, and angle-dependences of the sound generation.** (a) Power-dependence of sound signal generated by 70 fs pulses (with a 10 mm detecting distance and a 30° horizontal angle). (b) Distance-dependence of sound signal generated by 70 fs pulses (with a laser power of 1.38 W and a 40° horizontal angle). (c) Horizontal angle-dependence (with a laser power of 800 mW and a 10 mm detecting distance). (d) Vertical angle-dependence (with a laser power of 800 mW and a 10 mm measuring distance).

**SI3 PTA Sound generation efficiency**

Assuming spherical wave propagation, we describe the efficiency of the graphene sound generation as

$\eta\text{=}P_{rms}^{2}/(\rho_{g}v_{g}q_{0})$, (Eq. S2)

where $P_{rms}$ is the root-mean-square sound pressure, $\rho_{g}$= 1.16 kg/m^3^ is the air density, $v_{g}$ = 344 m/s is the sound velocity in air, $q_{0}$ is the input power. Taking $q_{0}$= 0.1 W and $P_{rms}$= 0.0696 Pa from Fig. 2a, we can obtain the sound generation efficiency, which is $\eta\text{=}$0.012 %. The efficiency of electrical generation of sound wave in the same sample is 0.010%. Compared with the PTA effect, the slightly lower efficiency can be attributed to the contact resistance energy dissipation.

**SI4 Effect of ambient gas molecule on anharmonic sound generation**

**Figure S2 The FFT of anharmonic acoustic wave generation of MLG in air and Helium gas, respectively.**

**SI5 PTA vs PA mechanisms**

We summarize the two aspects that can be used to distinguish between the PA and PTA mechanisms.

The peak width of the sound wave

|  | fs | ns | Mechanism Works?  (by comparison with experimental results) |
| --- | --- | --- | --- |
| PA | fs~ps  (The electron-gas molecule interaction has to be as fast as ~ps.) | 230ns  (The width is only limited by response time of instruments. Our system’s limitation is much higher than 6 kHz.) | **NO**  (We observed no fs~ps response, no 230 ns response, no oscilloscope-circuit limitation response. Rather, we observed a 6 kHz response.) |
| PTA | >> 230ns  (Since the TA sub-process is a thermal process, which takes long.) | >> 230ns | **YES** |

**Sound wave generation efficiency**

|  | fs | ns | Mechanism Works?  (by comparison with experimental results) |
| --- | --- | --- | --- |
| PA | High efficiency  (①The peak power is 1.8×10^6^ times higher than the ns pulse. So the photo-electron’s temperature, velocity, density are all much higher than those of ns pulse. It is therefore expected the electron-air molecule interaction is more extensive and effective.  ②Since e-phonon interaction takes ~ps, using fs laser pulses reduces thermal leakage to minimum.) | Low efficiency  (①Low peak power. Hence less extensive electron-air molecule interaction.  ②In PA mechanism thermal leakage does not produce any sound wave. A 230 ns pulse inevitably generates thermal leakage. Any thermal leakage will result in lower sound generation efficiency.) | **NO**  (We observed no difference in the sound generation efficiency.) |
| PTA | Even efficiency  (The TA sub-process is a thermal process, which takes much longer than both 130 fs and 230 ns.) | Even efficiency | **YES** |

**Table S1.** Distinguishing PA and PTA mechanisms using different ultrafast light pulses.

**SI6 The XRD characterization of our MLG sample**


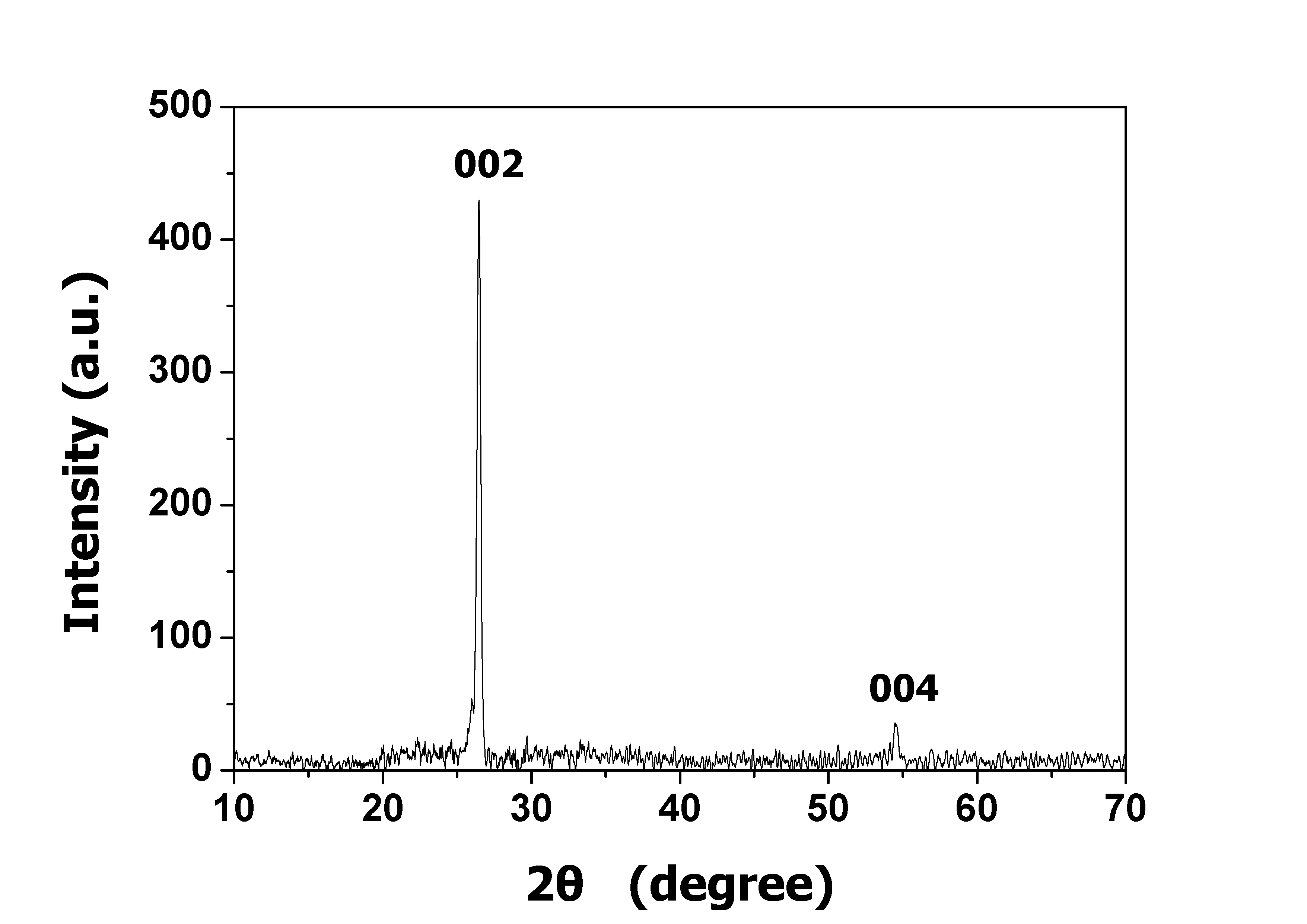


**Figure S3. X-ray diffraction pattern of our MLG sample.**
